# Prognostic factors for long-term improvement from stroke-related aphasia with adequate linguistic rehabilitation

**DOI:** 10.1007/s10072-019-03956-7

**Published:** 2019-06-10

**Authors:** Yoshitaka Nakagawa, Yoko Sano, Michitaka Funayama, Masahiro Kato

**Affiliations:** 1 Department of Rehabilitation, Edogawa Hospital, 2-24-18, Higashikoiwa, Edogawa-ku, Tokyo, 133-0052 Japan; 2 Department of Neuropsychiatry, Ashikaga Red Cross Hospital, Tochigi, Japan; 3 Department of Neurology, Edogawa Hospital, Tokyo, Japan

**Keywords:** Aphasia recovery, Long-term prognosis, Phonology, Semantics, Wernicke’s area, Age

## Abstract

In the past decade, several studies have reported potential prognostic factors for aphasia after stroke. However, these reports covered no more than 1 year after stroke onset, even though patients often continue to improve over longer periods. The present study included 121 patients with aphasia who received cognitive-based linguistic rehabilitation for at least 2 years post-onset. All were right-handed and had a lesion only in the left hemisphere. Aphasia outcome was predicted using multiple linear regression analysis. Age at onset, lesion in the left superior temporal gyrus including Wernicke’s area, and baseline linguistic abilities including aphasia severity and both phonological and semantic functions were significant predictors of long-term aphasia outcome. These findings suggest that the long-term outcome of aphasia following adequate linguistic rehabilitation can be predicted by age at onset, lesion area, and baseline linguistic abilities and that linguistic rehabilitation is particularly recommended for younger individuals with aphasia.

## Introduction

Recent studies have reported that age [1], initial aphasia severity [1–7], aphasia subtype [5], lesion location [8, 9], lesion volume [10], stroke severity [5, 11], and phonology [12] are potential prognostic factors for aphasia after stroke. Among these factors, initial aphasia severity has been proposed to be the most clinically relevant factor [7]. In these reports, however, recovery from aphasia covered no more than 1 year after onset [3–6, 11, 12], even though patients often continue to improve over longer periods. Kertesz and McCabe [2] found that certain patients with aphasia gradually improved over several years post-onset. Naeser et al. [13] found improvement in naming scores and phrase length in non-fluent speech 5 years post-stroke. Fitzpatrick et al. [14] reported that chronic aphasia patients continued to show improvements in picture-naming ability 5 to 15 years after stroke. Another limitation of the previous reports is that samples were mixed; i.e., they included patients who received linguistic therapy and others who did not. To address these shortcomings, we identified and enrolled patients with aphasia who received adequate linguistic rehabilitation for at least 2 years post-onset and carried out a multivariable regression analysis to determine prognostic factors for long-term improvement.

## Methods

### Subjects

Ethical aspects of this study were reviewed and approved by the Edogawa Hospital Human Research Ethics Committee. For our retrospective cohort study, patients with aphasia after stroke were recruited from Izunirayama Onsen Hospital and Edogawa Hospital between April 1975 and March 2015. To be included in the study, the participants (1) were native Japanese with ≥12 years of formal education, (2) were right-handed, (3) had stroke and stroke-related aphasia, (4) had a single lesion in the left hemisphere, (5) had no pre-stroke dementia, and (6) started a cognitive-based linguistic rehabilitation program within 6 months post-onset. Individuals were excluded if they (1) had any lesion in the right hemisphere, (2) stopped linguistic rehabilitation within year 2 post-onset, or (3) had developmental dyslexia or a psychiatric disorder that might interfere with aphasia assessment.

In total, 121 patients met these criteria, including 23 from Izunirayama Onsen Hospital and 98 from Edogawa Hospital. Among these patients, 93 were male and 28 female (Table 1). The proportion of males was high in this study, possibly reflecting the findings of previous reports that females with aphasia experience a significantly faster recovery than males [15, 16]. Among the 121 patients, 30 were university graduates and the remaining 91 were high school graduates. The etiology was either cerebral infarction (75 patients) or cerebral hemorrhage (46 patients). Average age at onset was 54.4 ± 11.9 years, and patients fell into the following age ranges: < 40 years, 10%; 40–49, 19%; 50–59, 35%; 60–69, 27%; and ≥ 70, 9%. Mean age in our cohort was lower than that in similar studies, probably because we included patients who underwent rehabilitation for at least 2 years post-onset. This is more likely to happen in younger patients, who have a strong motivation to complete lengthy rehabilitation protocols. The average time post-onset when patients started linguistic rehabilitation was 2.6 ± 1.5 months. Linguistic rehabilitation was performed using only cognitive-based linguistic therapy and focused on the main linguistic component(s) that were affected in each patient, e.g., phonology or semantics. Although linguistic rehabilitation tasks varied from patient to patient depending on their particular deficit(s) and aphasia severity, the standard rehabilitation protocol included comprehension tasks (picture/word matching task for both ideograms of Kanji (Chinese characters) and Kana (phonograms), sentence completion task, and auditory comprehension task), word retrieval (oral and written naming), description of scenes (oral and written description), reading aloud (Kanji written words, Kana written words, and sentences written in both Kanji and Kana), and a conversation task.

**Table 1:** Baseline characteristics of patients (*n* = 121)

Characteristic	Value
Demographics	
Mean age, years (SD)	54.4 (11.9)
Females, n (%)	28 (23)
Mean rehabilitation start, months post-onset (SD)	2.6 (1.5)
Right handedness, *n* (%)	121 (100)
Level of education, *n* (%)	
**≥** 16 years	30 (25)
**<** 16 years	91 (75)
Stroke subtype, *n* (%)	
Infarction	75 (62)
Intracerebral hemorrhage	46 (38)
Linguistic performance (SLTA)	
Mean baseline aphasia severity, score (SD) (0, worst; 10, best)	3.2 (2.6)

### Assessment

To assess baseline linguistic function, we administered the Standard Language Test of Aphasia (SLTA) [17], which has been widely used in Japan for nearly half a century and comprises 25 subtests of several language modalities, with four categories: auditory comprehension, speech, reading comprehension, and writing. To assess the baseline severity of overall aphasia, information acquired for the 25 subtests of the SLTA was summarized using a simpler scale, i.e., Guttman’s scalogram analysis or cumulative scaling [18]. Guttmann’s scalogram analysis is based on the deterministic model, and the subtests are ranked from least difficult to most difficult [18]. For example, if a patient received a passing mark of 80% in either the auditory sentence comprehension or reading sentence comprehension subtest, he or she was given one grade for a category requiring high-level comprehension. In this regard, there are 10 categories, each of which covers 1 to 3 subtests of the SLTA. Based on this analysis, the performance in the 25 subtests of the SLTA can be converted into a total score, which is then classified into 10 grades to assess aphasia severity, for which a grade of 0 reflects the poorest performance and a grade of 10 reflects the best [19].

Linguistic variables included the following six components: comprehension of ideogram (Kanji) written words, comprehension of written sentences mixed with ideograms (Kanji) and phonograms (Kana), word repetition, sentence repetition, oral reading of a single phonogram (Kana), and picture naming. The two kinds of written comprehension tasks were employed as a semantic factor. According to the principal component analysis for linguistic factors [20], the written comprehension task places more weight on semantics and less weight on phonology when compared with the spoken comprehension task at both the word and sentence levels, which also requires auditory phonological processing. In fact, spoken sentence comprehension task places more weight on phonology than on semantics [20]. The Japanese language offers an advantage for separately assessing semantics and phonology because its writing systems include ideograms of Kanji and Kana (phonograms) [21]. For Kana phonograms, the relationship between orthography and phonology is almost perfectly regular and governed by rules, suggesting that Kana reflects phonological function more than Kanji, especially when written as a single phonogram or in non-words. Considering these advantages, Kanji was employed for comprehension of written words to better assess the semantic factor, although the comprehension of Kanji also requires orthography. The other comprehension test for sentences involved both ideograms (Kanji) and phonograms (Kana) because Japanese sentences are always written using a mixture of both ideograms and phonograms, of which the former is mainly used for nouns or word roots and the latter for function words. With respect to the phonology factor, we employed word repetition, sentence repetition, and oral reading of a single phonogram (Kana). Based on Butler’s analysis [20], repetition is the most phonology-related task among the various linguistic tasks. As noted above, oral reading of a single phonogram (Kana) reflects phonological function, although it also involves recognition of a visually presented orthographic input. Picture naming was employed because word-finding difficulty is the hallmark of aphasia, although it depends on both phonological and semantic dysfunctions. Although syntactic components cannot be assessed independently, performance at the sentence level—but not the word level—requires syntactic processing along with semantic and phonological components.

Demographic variables included age at onset, gender, and education level. Clinical variables included stroke type, which was categorized as hemorrhage or infarction based on clinical examination by an experienced neurologist. Lesion location was assessed by a neuropsychiatrist (M.F.) who is experienced with this type of analysis and who had no prior knowledge of the patients’ linguistic performance. The assessment covered seven regions of interest that surround the left Sylvian fissure and are all related to linguistic function: Broca’s area and the precentral gyrus, the supramarginal and postcentral gyrus, the angular gyrus, the superior temporal gyrus that includes Wernicke’s area, the middle temporal gyrus, insula, and the basal ganglia. All major sulci in the lesions were identified via clinical MRI T1 or CT scans taken at onset. Lesions were located by taking into account the relation of the lesion boundary to the identified sulci. Subcortical lesions in white matter were included in the corresponding cortical lesions. Each area was assessed for the presence or absence of a lesion. Lesion volume was measured using the ABC/2 formula, where A represents the greatest lesion diameter based on MRI T1 or CT, B represents the diameter perpendicular to A, and C represents the approximate number of MRI T1 or CT slices through the lesion multiplied by the slice thickness. This ABC/2 method has excellent correlation with the planimetric method when applied to the measurements of intracerebral hemorrhage volumes [22] as well as ischemic stroke volume [23]. Because the sensitivity of MRI and CT differs, the use of different neuroradiological methods is a limitation of our study.

After patients had received linguistic rehabilitation for at least 2 years post-onset, aphasia outcome was estimated using the overall aphasia severity assessment in the SLTA at 2 years post-onset, which was applied to assess baseline aphasia severity. For all patients, the interval between linguistic assessments, i.e., as assessed with the SLTA, was more than 3 months, which was greater than the period for clinical practice effects [24] (Table 2)

**Table 2:** Potential prognostic variables for aphasia recovery

Demographics	
Age at onset	
Gender	
Education level	
Clinical variables	
Stroke type	
Baseline aphasia severity	
Lesion locations	
Broca’s area and the precentral gyrus	
The supramarginal and postcentral gyrus	
The angular gyrus	
The superior temporal gyrus which includes Wernicke’s area	
The middle temporal gyrus	
The basal ganglia	
Lesion volume	
Linguistic components	
Semantic factors	
Comprehension of ideogram (Kanji) written words	
Written-sentences comprehension mixed with ideograms (Kanji) and phonograms (Kana)	
Phonological factors	
Word repetition	
Sentence repetition	
Oral reading of a single phonogram (Kana)	
Both semantic and phonological factors	
Picture naming	

.

### Statistical analysis

First, we evaluated the influence of each explanatory variable, i.e., demographics (age, gender, and education level), stroke type, lesion volume, seven lesion locations, baseline aphasia severity, and the six baseline linguistic components, using univariate regression analysis. No single linguistic component had a correlation of more than 0.49 with other linguistic components, meaning these components are relatively independent of each other. The score for each subtest on the SLTA (which can range from 0 to 100%) was used for statistical analysis. Then, the combined influence of the variables on aphasia outcome was investigated using multivariable regression analysis. Variables with *P* < 0.01 in the univariate regression analysis were entered in the final multivariable linear regression model. A stepwise method was used to identify variables with the greatest influence on aphasia outcome. All analyses were performed using JMP v.11. The significance level for the multivariable linear regression model (two-tailed) was set at *P* < 0.05.

## Results

Out of a maximum score of 10 on the aphasia assessment, the average baseline aphasia severity score for the 121 patients was 3.2 ± 2.6, and the average aphasia outcome score was 6.2 ± 3.2. In the univariate regression analysis, four variables had *P* values of > 0.01 (indicating no statistical significance). Gender (*P* = 0.32), education level (*P* = 0.28), stroke type (*P* = 0.11), and lesion in the basal ganglia (*P* = 0.04) were excluded from the multivariable regression model. All other variables had *P* values of < 0.01.

**Fig. 1:**
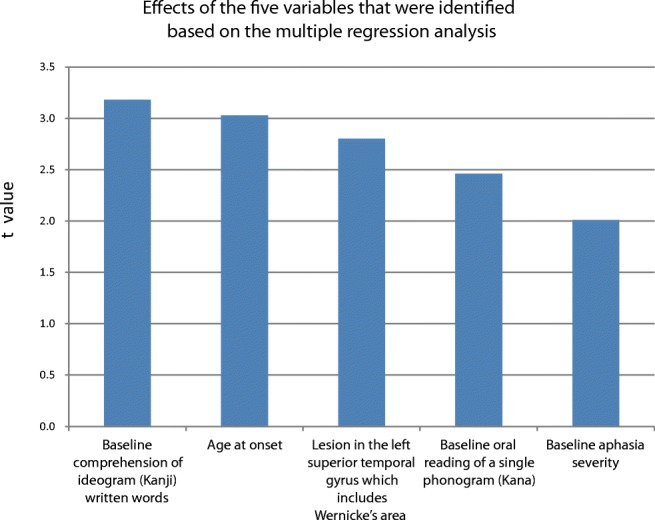
Effects of the five variables that were identified based on the multiple regression analysis

The stepwise method in the multivariable linear regression model revealed that age at onset, lesion in the left superior temporal gyrus (including Wernicke’s area), and baseline linguistic abilities (including aphasia severity and both phonological and semantic functions) were significant predictors of long-term aphasia outcome (Fig. 1). Regarding linguistic components, the result of the word-level or the syllable-level subtest—but not of the sentence-level subtest—was found to be a significant predictor, suggesting that semantic and phonologic functions rather than syntactic processing are key for predicting long-term aphasia outcome. Baseline comprehension of ideograms had the highest absolute *t* value (3.18), followed by age at onset (3.03), lesion in the left superior temporal gyrus including Wernicke’s area (2.80), baseline oral reading of phonograms (Kana) (2.46), and baseline aphasia severity (2.01). Aphasia outcome was predicted using the following formula.

$$ \mathrm{Aphasia}\ \mathrm{outcome}=4.989+\left[0.266\times \mathrm{baseline}\ \mathrm{aphasia}\ \mathrm{severity}\ \left(0\hbox{--} 10\right)\right]+\left[0.018\times \mathrm{baseline}\ \mathrm{oral}\ \mathrm{reading}\ \mathrm{of}\ \mathrm{phonograms}\ \left(\mathrm{Kana}\right)\ \left(0\hbox{--} 100\%\right)\right]+\left[0.029\times \mathrm{baseline}\ \mathrm{comprehension}\ \mathrm{of}\ \mathrm{ideogram}\ \left(\mathrm{Kanji}\right)\ \mathrm{written}\ \mathrm{words}\ \left(0\hbox{--} 100\%\right)\right]-\Big[0.050\times \left[\mathrm{age}\ \mathrm{at}\ \mathrm{onset}\ \left(\mathrm{years}\right)\right]-\left[0.667\times \mathrm{lesion}\ \mathrm{in}\ \mathrm{the}\ \mathrm{left}\ \mathrm{superior}\ \mathrm{temporal}\ \mathrm{gyrus}\ \mathrm{in}\mathrm{cluding}\ \mathrm{Wernicke}'\mathrm{s}\ \mathrm{area}\ \left(0\ \mathrm{or}\ 1\right)\right]. $$

Each variance inflation factor, which is a measure of the degree of multicollinearity of the independent variable with the other independent variables in the regression model, was less than 3.0, indicating that there was no significant multicollinearity. A prognostic model based on these five baseline predictors explained 55.6% of the observed variance. The results for this multiple linear regression model produced a *P* value of < 0.01.

## Discussion

Our results demonstrate that recovery from aphasia after stroke can be predicted by three attributes, namely, age at onset, lesion in the left superior temporal gyrus (including Wernicke’s area), and baseline linguistic abilities. This is the first report concerning the recovery from aphasia after stroke in a large sample of patients who underwent adequate, structured linguistic rehabilitation. Importantly, all previous studies used a relatively short follow-up period (< 1 year) [4, 12] and did not distinguish between patients who received linguistic therapy and those who did not [12].

Our results indicate that younger age at onset correlates with a better aphasia outcome. This finding is compatible with that of Laska et al. [1], who reported that only age at onset had a significant effect on aphasia recovery after multivariable regression analysis. However, our study was better able to evaluate the effect of age at onset because the mean age was 54.4 ± 11.9 years compared with 76 years in the Laska et al. study. Our study confirms the notion that younger individuals have greater potential for significant recovery from aphasia and suggests that linguistic rehabilitation should be a high priority for younger patients. Although the findings of certain other studies conflict with those of our present study [4, 25, 26], the follow-up duration of those studies was relatively short, i.e., ranging from only 10 days to 6 months post-onset. Our study is unique in that it combined younger patients with long-term follow-up, and this may underlie the significant age effect we observed.

In our study, the initial severity of aphasia was an independent predictor of recovery, and this is consistent with the results of previous studies [1–7]. In addition, our study demonstrated that baseline linguistic components of semantics and phonology rather than syntactic processing are related to long-term outcome of aphasia. According to Hachioui et al. [12], phonology is the strongest predictor among the three linguistic components, i.e., phonology, semantics, and syntactic processing. Indeed, our data support this conclusion. Regarding semantics, Hasegawa et al. [19] reported that improvement of comprehension underlies the improvement of other language components, e.g., speech, during recovery from aphasia, suggesting that baseline semantic function might also affect aphasia outcome.

The left superior temporal gyrus including Wernicke’s area was found to be closely related to aphasia outcome, as previous studies indicated [8, 9, 27, 28]. Wernicke’s area is involved in phonological processing [29–32] as well as semantic processing [30, 31, 33]. However, some researchers have questioned its role in auditory comprehension and have relegated its role to a certain aspect of auditory comprehension, i.e., the discrimination and recognition of speech sounds [32, 34]. Regarding the relationship between lesions in Wernicke’s area and the linguistic components we followed in our study, oral reading of phonograms might be partly related to the function of Wernicke’s area, as reported by Sakurai et al. [35], whereas comprehension of ideograms has been associated with the left posteroinferior temporal cortex [36].

Our study has several limitations that should be considered. First, there was selection bias, as patients who did not undergo linguistic rehabilitation and patients who quit linguistic rehabilitation within 2 years were not included. In addition, our study did not include a control group, i.e., aphasia patients who did not receive linguistic rehabilitation. This approach is necessary and appropriate, however, as the aim of the study was to investigate predictors of aphasia after adequate linguistic rehabilitation, and thus, we intentionally did not exclude older patients or those with severe aphasia. Second, although only cognitive-based linguistic therapy was provided for all patients, rehabilitation frequency was not controlled sufficiently. Third, the use of different neuroradiological methods, i.e., a mix of MRI and CT data, is a limitation of our study. Finally, there may be an inherent limitation in the linguistic-function data derived from the SLTA, which does not cover all linguistic functions that may be applicable to various social settings. This issue, however, also applies to other aphasia studies.

## Conclusions

Our study suggests that long-term aphasia outcome for patients who receive adequate linguistic rehabilitation for at least 2 years can be predicted by age at onset, lesion in the left superior temporal gyrus including Wernicke’s area, and baseline linguistic abilities. Our findings may enhance the accuracy of aphasia prognosis and inform rehabilitation strategies, and they suggest that adequate linguistic rehabilitation may particularly benefit younger individuals with aphasia.

## Abbreviations

SLTA: the Standard Language Test of Aphasia
